# Detection of Myoglobin with an Open-Cavity-Based Label-Free Photonic Crystal Biosensor

**DOI:** 10.1155/2013/808056

**Published:** 2013-06-02

**Authors:** Bailin Zhang, Juan Manuel Tamez-Vela, Steven Solis, Gilbert Bustamante, Ralph Peterson, Shafiqur Rahman, Andres Morales, Liang Tang, Jing Yong Ye

**Affiliations:** University of Texas at San Antonio, One UTSA Circle, San Antonio, TX 78249, USA

## Abstract

The label-free detection of one of the cardiac biomarkers, myoglobin, using a photonic-crystal-based biosensor in a total-internal-reflection configuration (PC-TIR) is presented in this paper. The PC-TIR sensor possesses a unique open optical microcavity that allows for several key advantages in biomolecular assays. In contrast to a conventional closed microcavity, the open configuration allows easy functionalization of the sensing surface for rapid biomolecular binding assays. Moreover, the properties of PC structures make it easy to be designed and engineered for operating at any optical wavelength. Through fine design of the photonic crystal structure, biochemical modification of the sensor surface, and integration with a microfluidic system, we have demonstrated that the detection sensitivity of the sensor for myoglobin has reached the clinically significant concentration range, enabling potential usage of this biosensor for diagnosis of acute myocardial infarction. The real-time response of the sensor to the myoglobin binding may potentially provide point-of-care monitoring of patients and treatment effects.

## 1. Introduction 

The diagnosis of cardiac disorders becomes more and more important with the incidence of acute myocardial infarction (AMI) commanding one of the highest mortality rates in the US and around the world [1]. Each year, approximately 635,000 people suffer from AMI [2], among whom it is estimated that 50% will die within the first hour of symptoms [3]. For this reason, many studies have been conducted to shorten the time required to diagnose AMI [2–5]. Given the complex pathophysiology of heart disease, interests have intensified in plasma biochemical markers to predict susceptibility and aid in patient management. After an AMI has occurred, cardiac biomarkers, such as myoglobin, troponin I (cTnI), troponin T (cTnT), and creatine kinase (CK-MB), are released into the bloodstream [3, 4, 6]. In 2000, the World Health Organization set a standard allowing physicians to use the troponins and CK-MB levels, in addition to ECG and the patients' history, to diagnose AMI [7]. Although the serum detection of these biomarkers aids in an accurate diagnosis, it is usually time consuming due to the laborious lab techniques and logistics of sample transportation to a central lab. Both the turnaround time for laboratory diagnosis and the elapsed time for cTnI or CK-MB biomarkers to be released into the body (up to 3 hours for cTnI and 6 hours for CK-MB after an AMI) [3] may lead to a delay in prime-time treatment or hospitalization of a patient with AMI [8]. Myoglobin, however, is one of the earliest biomolecules released into the bloodstream (~1 hour) after the AMI, reaches peak levels after 2 hours, and has been shown to be an early indicator of AMI [3, 6, 9]. Sallach et al. demonstrated that an increase of 20 ng/mL of myoglobin in 90 minutes provided a highly accurate diagnosis of AMI in patients with normal levels of cTnI [6]. Given that these cardiac markers have different characteristics, including clinical sensitivity and specificity, release time after symptom onset, clinical cutoff level (myoglobin 70–200 ng/mL; CK-MB 3.5–10 ng/mL; cTnI 0.06–1.5 ng/mL) [10, 11], and capability to remain elevated for a reasonable length of time, a rapid, accurate, and simultaneous measurement of the cardiac markers is important in reducing detection time, decreasing cost of patient treatment, and saving patient lives. This has led many researchers to study the use of label-free biosensors to detect myoglobin levels. 

Since the first discovery of cardiac biomarkers, many detection methods have been developed such as fluorescence immunoassay [12], surface plasmon resonance (SPR) sensor [13], resonant waveguide grating or quartz crystal microbalance [14], electrical signals from nanowire-based biosensors [15], microresonator based such as microcantilever biosensors [16, 17], and two-dimensional photonic-crystal biosensors [18–21]. For the fluorescence-based detection methods, despite its high sensitivity, the process of fluorescence labeling can be complicated and time consuming. The nanowire-based biosensors may offer label-free detection with high sensitivity, but measurement results are to be easily affected by pH values of solutions and charges of molecules [22], because the detection mechanism is based on the measurement of conductance change of a nanowire. For the cantilever-based resonator sensor, the out-of-plane vibration experiences a high viscous damping in liquid environment, thus lowering the Q-factor and mass resolution [23]. Although the SPR-based sensor has been successfully commercialized, it is expensive and has limited use for small molecular binding assays. In this paper, we report label-free bioassays of myoglobin with a novel photonic-crystal-based sensor having a unique open microcavity structure.

## 2. Material and Experimental Procedures 

### 2.1. Sensor Fabrication

The optical biosensor is designed based on a photonic crystal structure used in a total-internal-reflection configuration [24–28]. Briefly, the sensor is composed of a BK7 glass substrate, five alternating layers of two different dielectric materials (titania and silica), and a cavity layer at the surface. Each of the titania layer and the silica layer is 89.8 nm and 307.2 nm thick, respectively, and is fabricated on the substrate using a vacuum vapor deposition method, which is a well-established fabrication method and relatively inexpensive. The cavity layer of the sensor is formed with 382 nm of silica and 10 nm of silicon. The silicon layer is used to introduce an appropriate amount of absorption that produces a resonant dip in the reflectance spectrum of the sensor. A broadband light is introduced into the sensor substrate at an incidence angle of 64° through a prism. This PC-TIR sensor functions as a high-finesse Fabry-Pérot resonator, which enables it to yield a sharper resonance mode than SPR-based sensors and thus higher detection sensitivity, and yet the sensor surface available for analyte binding is open to free space and allows real-time binding measurements, bypassing the problems of porous structure-based biosensors. Our sensor is unique in the fact that it utilizes an open optical microcavity as opposed to a conventional closed optical microcavity. A traditional closed optical microcavity has a cavity layer sandwiched between two high-reflection surfaces. We create the open cavity by dividing the cavity layer of a traditional closed cavity in half, placing only one half into a TIR configuration. A microcavity is still created as the incident light is confined between the photonic crystal structure and its mirror image due to TIR. The open sensing surface is allows easy immobilization of analyte-recognition molecules on the surface and direct exposure of analyte molecules for real-time bioassays. When molecular bindings occur on the cavity layer surface, the wavelength of the resonant dip in the reflectance spectrum of the sensor shifts in a manner highly sensitive to analyte binding. Through monitoring the shift in the resonant dip, a real-time detection of the molecular binding can be performed. 

### 2.2. Biochemical Modification of Sensor Surface

Similar to the surface treatment protocol described in other papers [29–31], the sensor surface is cleaned and oxidized via immersion in a piranha solution—a mixture of sulfuric acid and hydrogen peroxide (98%-H_2_SO_4_ : 30%-H_2_O_2_ = 3 : 1) in a parafilm sealed beaker on a heated plate (80°C) for 1 hour followed by rinsing with a copious amount of deionized water for 2 minutes under sonication and then immersed in deionized water for overnight to completely remove the residue of acid. The sensor surface is further etched with trifluoroacetic acid (THF) for 90 minutes at room temperature. The THF residue is evaporated by putting the sensor in a vacuum chamber pumped overnight. The silanization of the sensor surface is finished by placing it in a freshly prepared 5% (v/v) 3-aminopropyltriethoxysilane (APTES) solution in 95% acetone for 10 minutes, followed by removing excess reaction reagents with acetone on a shaker for twelve times at 5 minute intervals. Finally, the curing of the silane linkage is carried out by drying the substrates on a hot plate at 110°C for 90 minutes. 

After the above process, the sensor surface is enriched with amine groups suitable for further conjugation with carboxyl-terminated biomolecules. Before the immobilization of cardiac myoglobin antibodies, the carboxyl methylated (CM) Dextran (150 kDa) is covalently conjugated onto the sensor surface via EDC/NHS chemistry to maximize the binding sites of cardiac myoglobin antibodies. The CM-Dextran (125 mg) is prepared in a HEPES (N-2-hydroxyethylpiperazine-N′-2-ethane sulfonic acid) buffer solution (2 mL, pH = 5.5). The carboxyl groups on the CM-Dextran are activated with the aid of 1-ethyl-3(3-dimethyl aminopropyl) carbodiimide (EDC) and N-hydroxysuccinimide (NHS) molecules for 10 minutes, with the molar ratios of –COOH : EDC :  NHS = 1 : 10 : 2.5. The CM-Dextran solution with activated carboxyl groups is adjusted to a pH value of 7.3 from 5.5 by adding an appropriate amount of NaOH solution. The activated CM-Dextran then reacts with the amine groups on the sensor surface for 4 hours at room temperature. The CM-Dextran immobilized on the surface is about 2.5 nm, confirmed by the sensor via the method described in Section 2.5. Finally, the immobilized CM-Dextran on the sensor surface is activated with a similar EDC/NHS chemistry and reacts with 200 *μ*L cardiac myoglobin antibodies (Fitzgerald Industrial, 10-M50C) to functionalize the sensor for specific detection of myoglobin (Fitzgerald Industrial, 30C-cp1030u). 

### 2.3. Preparation of a Microfluidic System

To load samples for analysis with the sensor, two microchannels having a width of 500 *μ*m, height of 380 *μ*m, and length of 5 mm are formed using a polydimethylsiloxane (PDMS) replica molding process. PDMS base and curing agents (Sylgard184, Dow Corning) are mixed at a ratio of 10 : 1. The mixture is degassed in a vacuum chamber for about 10 minutes and then cast on a mold and cured at room temperature. Finally, the two microchannels are sealed on the surface of the functionalized sensor. In order to inject the target sample solution or phosphate buffered saline (PBS) solution, Teflon tubings are used to connect the outlets of the microfluidic channels to a multichannel syringe pump. The inlets of the microfluidic channels are connected to two vials containing the target myoglobin samples and PBS solutions, respectively. The flow of cardiac myoglobin samples or buffer solutions onto the sensing areas is precisely controlled with the syringe pump. 

### 2.4. Experimental Setup Configuration

**Figure 1:**
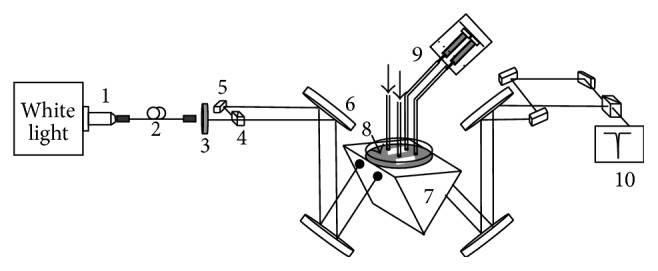
Schematic of experimental setup. A broadband white light source is coupled with an objective lens (1) into a single mode fiber (2). S-polarized light from a polarizer (3) goes through a beam splitter (4) and is directed via mirrors (5, 6) to a prism (7), which allows coupling the light into the sensor (8) at a 64° angle. Microfluidic channels attached to the sensor carry the sample solutions with controlled flow rates by a syringe pump (9). The reflected light from the sensor is detected by a high resolution spectrometer (10).

Our experimental setup is shown in Figure 1. A white light source is coupled into a single-mode optical fiber using an objective lens to obtain a good spatial mode. An aspherical lens is used to collimate the output light from the fiber as it passes through a linear polarizer to select s-polarization. The light is split into two parts and is delivered to the PC-TIR sensor substrate at an incident angle of 64° through a coupling prism. The two beams are carefully aligned to the center of the two microchannels on the sensor surface. The reflected light from the two microchannels on the sensor is collected using a high-resolution spectrometer (HR4000, Ocean Optics) where the resonant wavelength shifts are recorded in real time. One channel is used for flowing cardiac myoglobin samples, while the other is used as a reference channel to compensate for changes in the resonant wavelength due to mechanical drift or temperature fluctuations. 

### 2.5. Experimental Method

The myoglobin at various concentrations from 70 to 1000 ng/mL in PBS is measured using our PC-TIR sensor. Firstly, a PBS solution (pH = 7.4) is injected into both microchannels on the sensor surface. The resonant dip wavelengths in the reflectance spectra corresponding to the two channels are recorded as the detection baselines. Next, 200 *μ*L of cardiac myoglobin antibody solution (in PBS) is injected into one of the microchannels, replacing the PBS solution at a speed of 5 *μ*L/min. With the binding of the myoglobin antibody on the sensor surface, the resonant dip of the sensor shifts accordingly, which is a direct recording of the dynamic binding process. The sensor is then washed by making PBS flow through the both microchannels for 10 minutes at the same flow rate. An additional measurement of the resonant dip is taken after cleaning with PBS wash. The net shift of the resonant wavelength before and after the flow of cardiac myoglobin antibody solution reflects the amount of antibody molecules immobilized on the sensor surface. The binding thickness of the antibodies is confirmed by this method to be ~1 nm. The sensor is prepared for myoglobin assays due to the immobilization of the cardiac myoglobin antibodies on the sensor surface. A 200 *μ*L cardiac myoglobin solution in PBS is injected through one microchannel on the newly prepared sensor, while the other microchannel continuously maks PBS as a reference. Different concentrations of cardiac myoglobin samples are measured and the amount of cardiac myoglobin binding to the sensor surface is quantified by analysis of the corresponding resonant dip shift in the reflectance spectra. 

## 3. Results and Discussion

**Figure 2:**
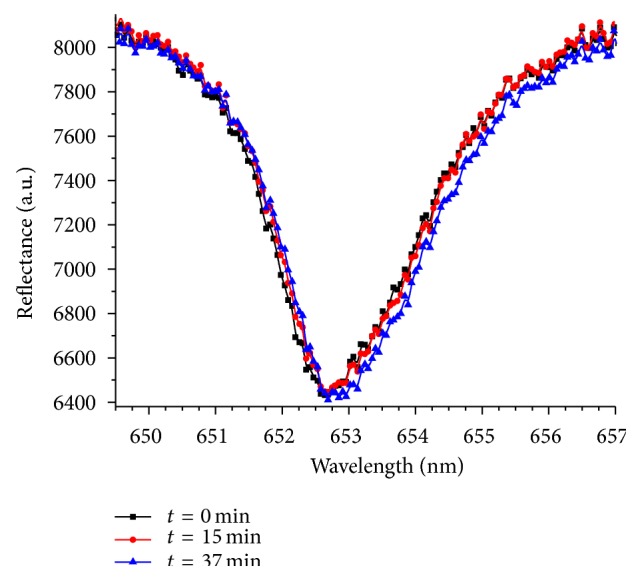
Typical reflectance spectra of a PC-TIR sensor when a 70 ng/mL myoglobin solution flows through the microchannel on the sensor surface. As the binding occurs on the surface, the resonant wavelength of the reflectance dip shifts to the longer wavelength with time. Three representative curves at times 0, 15, and 37 min are shown.

A series of the typical reflectance spectra of the sensor when making 200 *μ*L myoglobin solution (in PBS, 70 ng/mL) flow through a microchannel is shown in Figure 2. The full width at half maximum of the resonant dip is only 2.5 nm. The sharp resonant condition allows precise quantification of the center wavelength of the resonant dip. When the binding of myoglobin molecules to the immobilized antibodies occurs on the sensor surface, the resonant condition of the sensor changes due to an increase of the effective thickness of the cavity layer, thus producing a shift of the resonant wavelength. Although at the low concentration of myoglobin at 70 ng/mL the shift is minimal, it is still clearly measureable (Figure 2). The curves can be fitted with a Lorentzian function, which provides quantitative results of the shift of the center wavelength. At a constant concentration the shift increases with time, indicating that more myoglobin molecules are bound to the antibodies on the sensor surface. When the concentration of myoglobin increases, the shift of the resonant wavelength also increases and can become much bigger than that shown in Figure 2. 

**Figure 3:**
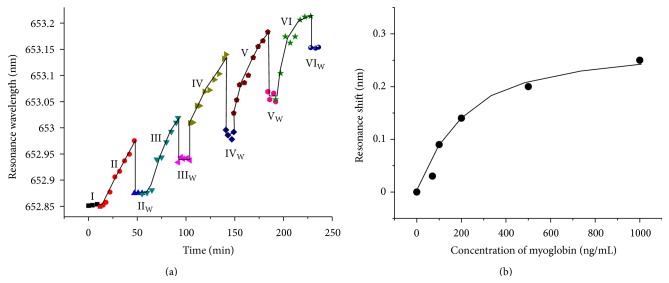
(a) Binding kinetics of myoglobin on a functionalized PC-TIR sensor surface. Before detection, a detection baseline is first recorded by making PBS flow through a microchannel on the sensor surface (I). Stages II–VI show the results of binding assays of myoglobin with concentrations of  70, 100, 200, 500, and 1000 ng/mL, respectively. The remaining captured myoglobin on the sensor surface after washing with PBS is then recorded as Stages II_W_–*VI*_W_. (b). Resonant wavelength shift of the sensor as a function of myoglobin concentrations.

The binding kinetics of myoglobin with its antibody is illustrated in Figure 3(a). The time-dependent wavelength shift of the sensor resonant dip is caused by the interaction events between myoglobin and the antibodies immobilized on the sensor surface. The time-dependent response is close to linear for the first concentration of 70 ng/mL. The linear relationship is because the binding sites available on the sensor surface are much larger than the number of myoglobin molecules. Over the time, the available interaction sites decrease until all of the binding sites are occupied, resulting in a saturation of the binding curve for the high concentration (1000 ng/mL). After binding of myoglobin at each concentration, the sensor surface is washed with PBS and a drop of the resonant wavelength is observed, which can be attributed to partial removal of loosely bound molecules. The end point of the resonant wavelength shift after PBS wash is plotted as a function of myoglobin concentrations in Figure 3(b). Saturation of binding has been observed at higher myoglobin concentrations. Based on our transfer matrix simulation of multilayer interference in our sensor [27], the final resonant wavelength shift of 0.25 nm for 1000 ng/mL of myoglobin corresponds to a binding thickness of myoglobin of 0.30 nm. Our experimental result indicates that a PC-TIR can be functionalized for sensitive detection of myoglobin with a concentration ranging from 70 to 1000 ng/mL. This sensing range of myoglobin meets the present clinical diagnostic requirement for myocardial damage that results in an elevation of myoglobin to more than 110 ng/mL [32, 33].

**Figure 4:**
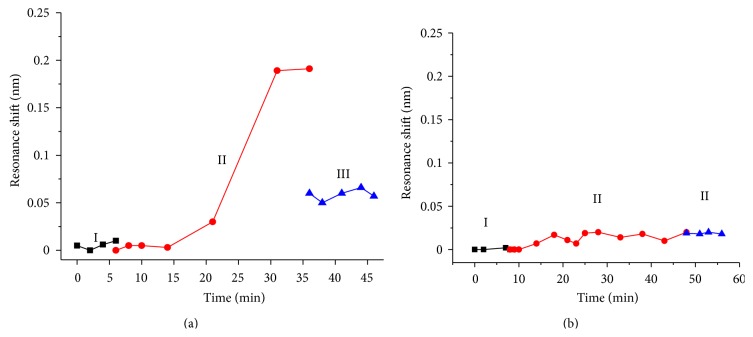
Binding kinetics of myoglobin having concentrations of 1000 ng/mL (a) and 70 ng ng/mL (b) in PBS, respectively, on a sensor surface without immobilized antibodies. Stage I: PBS baseline; II: myoglobin solution flowed through the sensing area; III: the remained myoglobin after PBS wash.

In order to confirm that the resonant wavelength shift is caused by specific interactions between the antibodies and myoglobin, we conducted a control experiment. For that, myoglobin solutions with the highest (1000 ng/mL) and lowest (70 ng/mL) concentrations used in the binding assay experiment were selected to be injected into a microchannel on a sensor surface without immobilized antibodies. As demonstrated in Figure 4, after PBS wash the average resonant wavelength shift is only ~0.050 (Figure 4(a)) and 0.018 nm (Figure 4(b)), respectively. This result indicates that there is minimal nonspecific binding for a sensor without the immobilization of antibodies, while the resonant wavelength shift for a sensor functionalized with antibodies is caused by specific binding between myoglobin and the antibodies. 

**Figure 5:**
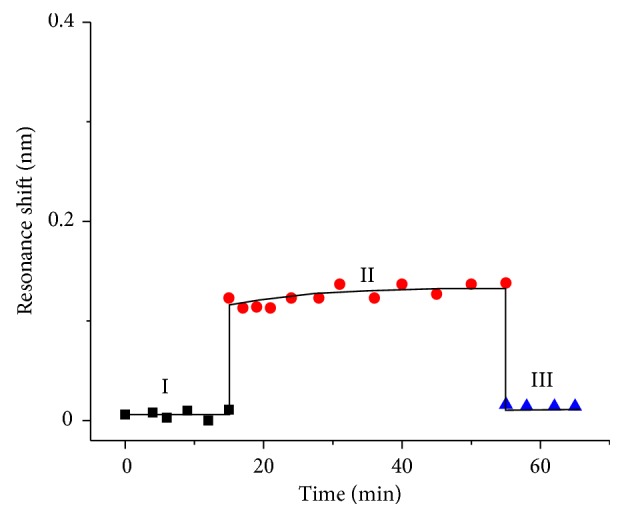
Binding kinetics of cTnI on a PC-TIR sensor with immobilized myoglobin antibodies. Before cTnI was injected, a detection baseline was first recorded by making PBS flow through a microchannel on the sensor surface (Stage I). Stage II shows the sensor response when 200 *μ*L of cTnI with a concentration of 200 ng/mL was flowing on the sensor surface. Stage III shows that the resonant wavelength returned to the baseline after a PBS wash.

To further test the specificity of the sensor to myoglobin, we carried out another control experiment by checking the binding of unrelated protein molecules with the sensor having immobilized myoglobin antibodies. For that, cardiac troponin I (cTnI) was chosen as a random protein molecule. A solution of 200 *μ*L cTnI with a concentration of 200 ng/mL in PBS was flowed across the sensor surface immobilized with myoglobin antibodies. Although we observed a jump of the resonant wavelength when starting injection of cTnI onto the sensor surface, the wavelength shift returned to the baseline level after the sensor was washed with PBS (Figure 5). The initial jump of the resonant wavelength can be attributed to the change of the bulk refractive index caused by replacing PBS with the cTnI solution. Except for the initial jump, the flat sensor response during this period indicated that no real binding occurred when cTnI was flowing on the sensor surface. The fact that the final wavelength shift returned to the baseline after the sensor was rinsed with water further proved that there was no binding of cTnI to the sensor functionalized for myoglobin assays. Therefore, this experimental result confirms the specificity of our sensor for detecting the myoglobin cardiac biomarker. 

Besides quantifying myoglobin with different concentrations, the PC-TIR sensor can also be used to obtain the dissociation constant *K*_*d*_ at equilibrium, which is an important parameter in evaluating the biochemical binding activities. Assuming a simple biomolecular binding model where two binding components A and B form a binding complex as AB, [A] + [B] *⇋* [AB], the kinetic rate constants are described as follows [34]:

(1) dRdt=kon·C(Rmax−Rt)−koff·Rt,

where *R* is the response of the sensor, while *R*_*t*_ and *R*_max_ are the response at time *t* and the maximum binding response, respectively. *C* is the concentration of analyte in solution. *k*_*on*_ and *k*_*off*_ are the binding and dissociation rate constants, respectively. At equilibrium, one obtains

**Figure 6:**
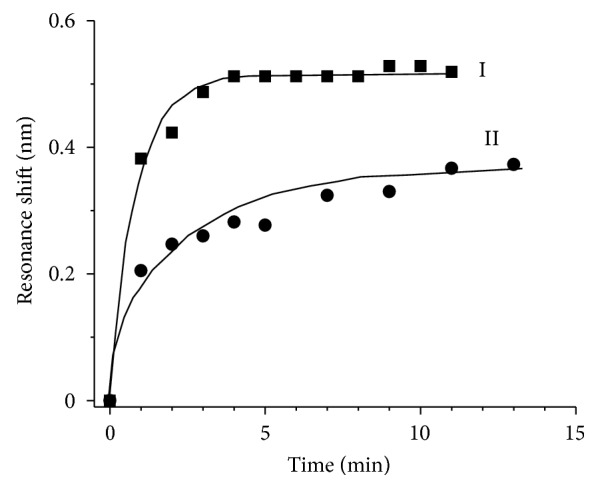
Kinetic binding shift curve of myoglobin (I: 8 ug/mL and II: 2.6 ug/mL in PBS solution) on PC-TIR surface immobilized with myoglobin antibody.

(2) KdReq=C(Rmax−Rt),

where *K*_*d*_ is the equilibrium dissociation constant, *K*_*d*_ = *k*_*off*_/*K*_*on*_. *R*_eq_ is the sensor response at equilibrium corresponding to a concentration of *C*. By measuring the resonance shifts at equilibrium for two different concentrations, one can calculate *K*_*d*_ by solving (2). To ensure binding equilibrium for calculating the equilibrium dissociation constant *K*_*d*_, we used myoglobin samples at two high concentrations, 8000 ng/mL and 2600 ng/mL, for the binding assays with our PC-TIR sensor. The binding curves are plotted in Figure 6, which shows that equilibrium has been reached at two levels corresponding to the two concentrations used. From the sensor responses at the equilibrium levels and based on (2), the *K*_*d*_ of myoglobin bound with antibodies on the PC-TIR sensor surface was calculated to be 1.2 nM, which is in good agreement with the value of 1.3 nM reported previously in [35]. 

It should be noted that myoglobin is not specifically indicative of acute myocardial infarction, because it is also present in skeletal muscle; however, it may still provide the earliest indication of myocardial injury and other valuable prognostic information when it is used in combination with other biomarkers [36]. cTnI is the most widely used biomarker, due to its nearly complete cardiac tissue specificity and sensitivity. They remain elevated for 4–10 days after the onset of AMI, indicating their capability to remain elevated for a reasonable length of time to allow a suitable diagnostic window. However, since it takes approximately 4 hrs for cardiac troponins to reach detectable concentrations in blood, they cannot be considered as early markers. On the other hand, myoglobin is one of the earliest biomarkers released into blood circulation after AMI. However, myoglobin is rapidly cleared out from blood compared to other cardiac biomarkers, limiting its diagnostic usefulness in patients after 8–12 hours of presenting symptoms. A combination blood test of cTnI and myoglobin may allow the achievement of high diagnostic sensitivity and specificity [4]. A sensitive biosensor is needed for rapid detection right after the onset of the elevation of myoglobin concentrations. A PC-TIR sensor may potentially address this unmet demand, since it can offer sensitive and rapid detection of myoglobin in the clinically significant concentration range as demonstrated in this study. 

## 4. Conclusion

A photonic crystal structure used in a TIR configuration can form a sensitive biosensor with a unique open microcavity as its sensing surface. Such a sensor is successfully fabricated at low cost and applied for the label-free binding assay of myoglobin for potential early diagnosis of myocardial infarction. The sensor detection limit is as low as 70 ng/mL, which falls in the clinical diagnostic level of AMI patients. The experimental results reported here serve as a stepping stone towards potential applications of the PC-TIR sensor for earliest diagnosis and identification of AMI patients. In the future, more experiments with the PC-TIR sensor will include the use of other cardiac biomarkers along with actual blood samples.
